# Mental distress and virtual mental health resource use amid the COVID-19 pandemic: Findings from a cross-sectional study in Canada

**DOI:** 10.1177/20552076231173528

**Published:** 2023-05-04

**Authors:** Trevor Goodyear, Chris Richardson, Bilal Aziz, Allie Slemon, Anne Gadermann, Zachary Daly, Corey McAuliffe, Javiera Pumarino, Kimberly C Thomson, Emily K Jenkins

**Affiliations:** 1 School of Nursing, 8166 University of British Columbia, Vancouver, Canada; 2 School of Population and Public Health, 8166 University of British Columbia, Vancouver, Canada; 3 Centre for Health Evaluation and Outcome Sciences, St Paul's Hospital, Vancouver, Canada; 4 School of Nursing, 8205 University of Victoria, Victoria, Canada; 5 Human Early Learning Partnership, School of Population and Public Health, University of British Columbia, Vancouver, Canada

**Keywords:** Mental health, psychological distress, digital health, virtual health, virtual mental health, health communications, health literacy, COVID-19, utilization, access

## Abstract

**Objective:**

This paper characterizes levels of mental distress among adults living in Canada amid the COVID-19 pandemic and examines the extent of virtual mental health resource use, including reasons for non-use, among adults with moderate to severe distress.

**Methods:**

Data are drawn from a cross-sectional monitoring survey (29 November to 7 December 2021) on the mental health of adults (*N*  =  3030) in Canada during the pandemic. Levels of mental distress were assessed using the Kessler Psychological Distress Scale. Descriptive statistics were used to examine virtual mental health resource use among participants with moderate to severe distress, including self-reported reasons for non-use.

**Results:**

Levels of mental distress were classified as none to low (48.8% of participants), moderate (36.6%), and severe (14.6%). Virtual mental health resource use was endorsed by 14.2% of participants with moderate distress and 32% of those with severe distress. Participants with moderate to severe distress reported a range of reasons for not using virtual mental health resources, including not feeling as though they needed help (37.4%), not thinking the supports would be helpful (26.2%), and preferring in-person supports (23.4%), among other reasons.

**Conclusions:**

This study identified a high burden of mental distress among adults in Canada during the COVID-19 pandemic alongside an apparent mismatch between actual and perceived need for support, including through virtual mental health resources. Findings on virtual mental health resource use, and reasons for non-use, offer directions for mental health promotion and health communication related to mental health literacy and the awareness and appropriateness of virtual mental health resources.

## Introduction

Mental health challenges are a leading cause of health-related burden globally.^1^ The ongoing COVID-19 pandemic and associated public health measures have contributed to this burden, as evidenced by reports of widespread, adverse impacts to population mental health since the onset of the pandemic.^2,3^ This includes elevated levels of negative emotions, such as fear, stress, worry, loneliness, and irritability,^4–6^ alongside increased rates of depression, anxiety, post-traumatic stress symptoms, and suicidal thoughts.^7–10^ A recent systematic review and meta-analysis quantifying the mental health impacts of COVID-19 in 204 countries identified that the global prevalence of major depressive disorder has increased by 28%, together with a 26% increase in the prevalence of anxiety disorders.^9^

Among the suite of interventions available to support mental health and wellbeing are virtual mental health resources. These refer to apps, website, online tools, or other web-based supports used in the realm of digital mental health care, which can involve a mix of synchronous (i.e. in real time) telephone/video visits with healthcare providers, and asynchronous tools, such as online self-management or skill-building interventions.^1,11,12^ While not intended to fully replace in-person services at the systems level, virtual mental health resources have proven to be safe, effective, and adaptable tools to support people experiencing mental health challenges.^13–16^ As we have previously suggested,^11^ these resources are relevant to people across the continua of mental health and illness, including those who experience mild to moderate, or even “sub-clinical” symptoms of stress, anxiety, and low mood, as well as those who have more severe mental distress and/or illness. Virtual mental health resources are becoming increasingly recognized, as is virtual healthcare more generally. Indeed, the World Health Organization recently underscored that digital health promotion strategies can “benefit people in a way that is ethical, safe, secure, reliable, equitable and sustainable.”^17^ Amid this digital health era, growth in provision of and need for virtual mental health resources has ignited following the escalation of the COVID-19 pandemic. This was especially so after many in-person services were reduced, suspended, or transitioned online early on in the pandemic, and as quarantine and physical distancing measures were introduced.^16,18,19^ Considering the population-level mental health burden of the pandemic, which continues to unfold internationally, virtual mental health resources have the potential to offer a scalable means of promoting stress management, skills building, and healthy coping among people experiencing or “at-risk” of mental health challenges.^17^ This includes those who have not previously accessed such supports but may benefit from doing so.^17^

In many settings, uptake of virtual mental health resources has lagged behind their suggested acceptability and potential. A 2018 study of psychologists in the United States found that although 74% of participants viewed telehealth as a useful means of mental health intervention, only 26% had used it in their practice.^20^ Moreover, while virtual mental health resource use and capabilities have grown over time,^16^ their uptake—and, thus their potential impact—continues to be low in many contexts. In our 2020 survey of a nationally representative sample of 3000 adults living in Canada, we determined the prevalence of asynchronous virtual mental health resource use to be very low among both the general population (2.0%), and those who reported adverse mental health impacts related to the COVID-19 pandemic (2.8%), among whom we also examined associations between sociodemographic and health-related characteristics and resource uptake.^11^ Similarly, a 2020 study conducted in the United Kingdom (UK) with 26,720 adults found that only 8% had used phone helpline or online services to support their mental health,^21^ despite a high burden of mental health morbidity in the UK during the early phase of the pandemic.^22^ Fortunately, more recent work has begun to show an uptick in virtual mental health resource use in the pandemic context,^23,24^ highlighting the potential value and scalability of these supports during periods of high demand. Still, there remain opportunities for research examining population-level extent of use (and non-use) of virtual mental health resources. This is an especially salient area of inquiry given that the pandemic is continuing to threaten population mental health and that governmental and other stakeholders are investing in virtual mental health care infrastructure.^16,25^ Building on our earlier work in this area,^11^ this study draws on the most recent round of data from a repeated, cross-sectional monitoring survey to provide an extended analysis of population mental health and virtual mental health resource use in Canada amid the pandemic. The objectives of this study are to characterize levels of mental distress among adults living in Canada in the context of the COVID-19 pandemic and to examine the extent of virtual mental health resource use, including reasons for non-use, among adults experiencing moderate to severe levels of mental distress.

## Methods

### Study overview

This study stems from a partnership between our research team at the University of British Columbia and the Canadian Mental Health Association. Together, we are leading a repeated cross-sectional monitoring survey, “*Assessing the Impacts of COVID-19 on Mental Health*.” This survey has been delivered four times between 2020 and 2021 and is set in Canada, where, as of 2020, 92% of people report using the internet.^26^ Items for this survey were first developed in 2020 by the Mental Health Foundation in the UK, and are informed by research into the mental health impacts of past pandemics, as well as input from people with lived and living experience of mental health challenges, gathered via a participatory citizens’ jury.^27^ The survey was made available in both French and English, with some items adapted to facilitate the conduct of additional analyses and to support use in the Canadian context; other scales were added without adaptation, including the 6-item version of the Kessler Psychological Distress Scale (K6). Approval for this study was obtained from the University of British Columbia Behavioural Research Ethics Board (#H20-01273).

### Data collection

A national polling vendor, Maru/Matchbox, distributed our anonymous online survey to members of “Maru Voice Canada.”^28^ This panel is an online research community of approximately 125,000 adults (18 years or older) living across Canada who have declared that they will take part in future surveys, if invited. Panel members were randomly selected to receive an invitation to participate in the study. Selection was stratified according to Canadian Census-informed socio-demographic characteristics—including age, gender, annual household income, province/territory—and adjusted for response propensity to yield a sample that is nationally representative according to these characteristics. To further support the representativeness of the sample, the polling vendor included statistical weights developed using current census data for age, gender, province/territory, and annual household income in the adult population of Canada.

Data were drawn from the fourth round of this repeated cross-sectional survey, which was open between 29 November and 7 December 2021. This data collection period corresponded with a time during which many Canadian provinces began to experience an uptick in COVID-19 case counts, heading into the winter holidays,^29^ leaving many people apprehensive about COVID-19 warnings and associated public health projections in the year to come. This period also coincides with changes to healthcare provider reimbursement for virtual care in many Canadian provinces and territories, including a loosening of provider billing regulations and increase in virtual service use in conjunction with the pandemic.^30,31^ Of note, Canada has a public healthcare system wherein most health services are directly funded by government, not individuals. The invitation-to-response rate for this survey round was 30%, which is consistent with our prior rounds in 2020–2021 (32%, 36%, and 33%). The maximum margin of error for proportions derived from a survey sample such as ours is  + /−1.79% at a 95% level of confidence. Participants provided online informed consent and received a small honorarium from Maru/Matchbox.

### Measures

#### Demographics

Standard self-reported sociodemographic assessments included age in years, current gender, sex assigned at birth, annual household income, highest level of education completed, and ethno-cultural origin, which we collapsed into Indigenous, racialized (non-Indigenous), and non-racialized.

#### Mental distress

The K6 was used to assess mental distress experienced over the prior 30 days.^32^ More specifically, the K6 items ask participants to think back over the past 30 days and rate how often they felt nervous, hopeless, restless or fidgety, worthless, depressed, or that everything was an effort. The response options for each of the six assessments are: “none of the time” (0 points), “a little of the time” (1 point), “some of the time” (2 points), “most of the time” (3 points), and “all of the time” (4 points). Responses to the six questions are summed to create a total score that ranges between 0 and 24, with higher scores indicating greater distress. Total scores on K6 can also be categorized as “Low to none” (0-4 points), “Moderate” (5-12 points), and “Severe” (13 points or higher), up to a total possible score of 24 points.^33^

#### Use of virtual mental health resources

The use of virtual mental health resources was assessed by asking participants, “If you have experienced mental health challenges at any point during the pandemic, have you used virtual (online or phone-based) mental health services and supports (e.g. counselling, mental health coaching sessions)?” Response options were “Yes,” “No,” “Not applicable. I haven’t experienced a mental health challenge during the pandemic,” and “Prefer not to say.” Participants who answered “No” were also asked, “Virtual mental health supports are receiving substantial investment to help people cope with mental health challenges during the pandemic. In order to inform better programs, could you please indicate why you did not access virtual mental health supports? (Select all that apply).” This question included a checkbox list of responses: “Stigma (I was afraid of what others would think of me),” “Didn’t feel I needed help,” “Privacy concerns,” “I don’t think they would be helpful,” “I didn’t know these supports were available,” “Necessary equipment not available (e.g. computer, smart phone),” “Connectivity issues (e.g. no/slow internet connection),” “Competing demands on my time,” “I prefer in-person health care supports,” and “Other (Please specify).” These response options were developed based on our team's prior clinical and research experiences in mental health, as well as our literature review for a related study.^11^

### Statistical analyses

Descriptive statistics were used to characterize the sample as a whole, and across each of the following categories of mental distress, based on K6 scores: None to low (0–4 points), Moderate (5–12 points), and Severe (13 points or higher). Descriptive statistics (proportions and chi-square test where indicated) were also used to determine the proportion of respondents experiencing moderate to severe levels of distress who reported that they did or did not access virtual mental health resources, along with their indicated reasons for not accessing virtual mental health resources. All analyses were conducted using the data with statistical weights applied.

## Results

**Figure 1.**
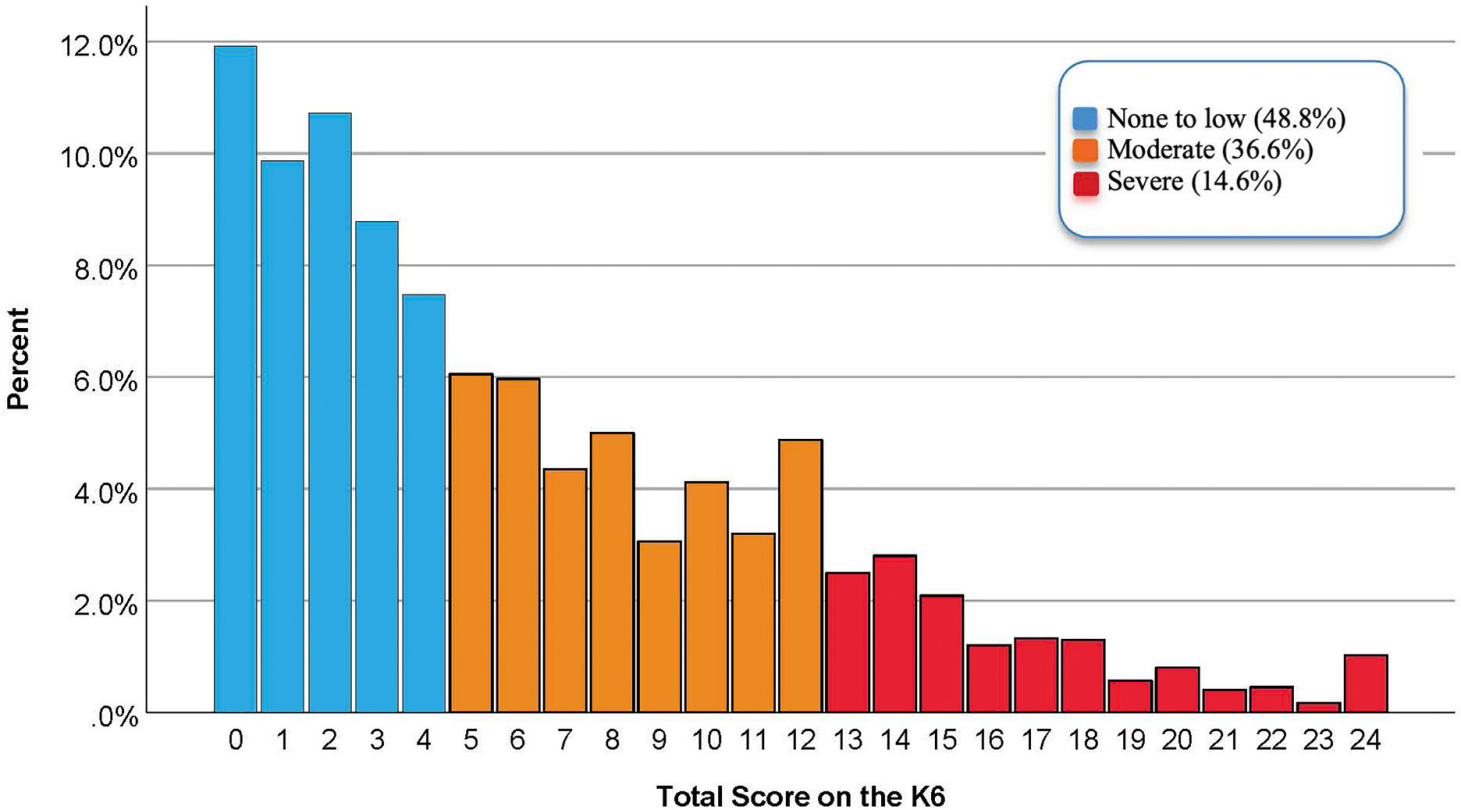
Distribution of total scores on the K6 grouped according to severity of distress.

Detailed sociodemographic characteristics of the sample of 3030 are presented in Table 1, as are differences in K6 mental distress category by these demographics. For example, this table reports higher levels of distress among people with pre-existing mental health challenges (relative to those without), women and non-binary people (relative to men), Indigenous Peoples (relative to other racialized and non-racialized people), and people with lower annual household incomes. Overall, total scores on the K6 ranged from 0 to 24 and had a mean of 6.3 (SD 5.6). The distribution of total K6 scores grouped by category of severity is presented in Figure 1. None to low levels of distress were experienced by 48.8% of participants, while 36.6% were classified as experiencing moderate levels, and 14.6% were classified as experiencing severe levels.

**Table 1.** Sociodemographic characteristics of the sample.

	K6 distress category			Total *n* (%)
Demographic	None to low *n* (%)	Moderate *n* (%)	Severe *n* (%)	
Age in years				
18–34	249 (29.5)	370 (43.8)	226 (26.7)	845 (27.9)
35–54	473 (44.6)	419 (39.5)	169 (15.9)	1061 (35.0)
55 +	755 (67.2)	321 (28.6)	48 (4.3)	1124 (37.1)
Gender				
Cisgender man	799 (54.1)	514 (34.8)	165 (11.2)	1478 (48.8)
Cisgender woman	672 (44.8)	577 (38.5)	251 (16.7)	1500 (49.5)
Transgender woman	1 (11.1)	3 (33.3)	5 (55.6)	9 (0.3)
Transgender man	1 (16.7)	1 (16.7)	4 (66.7)	6 (0.2)
Non-binary	1 (4.3)	10 (43.5)	12 (52.2)	23 (0.8)
Two-Spirit	0 (0)	0 (0)	2 (100)	2 (0.1)
Not listed	0 (0)	1 (50.0)	1 (50.0)	2 (0.1)
Household income (annual), CAD				
Under $25k	75 (34.4)	84 (38.5)	59 (27.1)	218 (7.5)
$25k–<$50k	221 (43.6)	183 (36.1)	103 (20.3)	507 (17.5)
$50k–<$100k	472 (49.4)	356 (37.2)	128 (13.4)	956 (33.0)
$100k +	655 (53.9)	427 (35.1)	133 (10.9)	1215 (42.0)
Education completed				
High school or less	249 (52.6)	158 (33.4)	66 (14.0)	473 (15.6)
Some college or university	278 (53.1)	159 (30.3)	87 (16.6)	524 (17.3)
College or university graduate	951 (46.8)	793 (39.0)	290 (14.3)	2034 (67.1)
Race/ethnicity^a^				
Non-racialized	1013 (51.4)	718 (36.4)	240 (12.2)	1971 (68.0)
Racialized (non-Indigenous)	364 (44.8)	289 (35.6)	159 (19.6)	812 (28.0)
Indigenous	38 (33.0)	51 (44.3)	26 (22.6)	115 (4.0)
Urban/rural				
Urban	677 (46.5)	542 (37.2)	237 (16.3)	1456 (48.1)
Rural	302 (54.0)	186 (33.3)	71 (12.7)	559 (18.4)
Sub-urban	498 (49.1)	382 (37.6)	135 (13.3)	1015 (33.5)
Pre-existing mental health condition				
No	1390 (57.4)	807 (33.3)	226 (9.3)	2423 (80.8)
Yes	84 (14.6)	284 (49.4)	207 (36.0)	575 (19.2)
Total	1478 (48.8)	1110 (36.6)	443 (14.6)	3030 (100.0)

Due to a small amount of missing data, the total *n* for some sociodemographic characteristics is lower than 3030.

^a^ Participants who identified only European origins were classified as non-racialized, those who identified one or more non-European origins were classified as racialized persons, and those who identified Indigenous origins were classified as Indigenous, regardless of other reported origins.

Use of virtual mental health services during the pandemic by category of mental distress is presented in Table 2. There was a significant relationship between the use of virtual mental health services and mental distress categories (*X^2^* (4, *N*  =  2990)  =  869, *p* < 0.001), with greater use of virtual resources among those reporting more severe levels of distress. Of survey respondents in the none to low category of distress, only 3.3% reported using virtual mental health services; the majority (69.7%) reported that this question was not applicable because they had not experienced a mental health challenge during the pandemic. Use of virtual mental health services was reported by 14.2% of respondents in the moderate category of distress and 32.0% of those in the severe category.

**Table 2.** Use of virtual mental health services and supports by level of mental distress.

	Use of virtual mental health services *n* (%)			Total *n* (%)
Mental distress category	Yes	No	Not applicable	
None to low	48 (3.3)	399 (27.1)	1026 (69.7)	1473 (49.3)
Moderate	154 (14.2)	637 (58.9)	291 (26.9)	1082 (36.2)
Severe	139 (32.0)	272 (62.5)	24 (5.5)	435 (14.5)
Total *n* (%)	341 (11.4)	1308 (43.7)	1341 (44.8)	2990 (100.0)

*Note*: There was a significant relationship between use of virtual mental health services and mental distress categories (*X*^2^ (4, *N* = 2990) = 869, *p* < 0.001).

The reasons for not using virtual mental resources among those participants who experienced moderate to severe levels of distress and reported having not used these resources are presented in Table 3. Individual reasons for not using virtual services were grouped into the following categories: Perceptions about resource availability and appropriateness; time-, privacy-, and stigma-related concerns; and Internet and technology access. The three most frequently reported reasons for not using virtual mental health resources were: “Didn’t feel I needed help” (37.4%), “I don’t think they would be helpful” (26.2%), and “I prefer in-person health care supports” (23.4%).

**Table 3.** Reasons for not using virtual mental health services by category of mental distress.

	Category of mental distress		
Reason for not using virtual services	Moderate *n* (% of 637 respondents with moderate distress)	Severe *n* (% of 272 respondents with severe distress)	Moderate–severe n (% of 909 respondents with moderate & severe distress)^a^
*Perceptions about resource availability and appropriateness*			
Didn’t feel I needed help	284 (44.6)	56 (20.6)	340 (37.4)
I don’t think they would be helpful	154 (24.2)	84 (30.9)	238 (26.2)
I prefer in-person health care supports	151 (23.7)	62 (22.8)	213 (23.4)
I didn’t know these supports were available	105 (16.5)	57 (21.0)	162 (17.8)
*Time-, privacy-, and stigma-related concerns*			
Competing demands on my time	75 (11.8)	52 (19.1)	127 (14.0)
Privacy concerns	58 (9.1)	41 (15.1)	99 (10.9)
Stigma (I was afraid of what others would think of me)	35 (5.5)	38 (14.0)	73 (8.0)
*Internet and technology access*			
Connectivity issues (e.g. no/slow internet connection)	24 (3.8)	11 (4.0)	35 (3.9)
Necessary equipment not available (e.g. computer, smart phone)	16 (2.5)	9 (3.3)	25 (2.8)

^a^ The number and percentages presented in this column total 1312 (144%) because participants could select more than one reason for not using virtual services.

## Discussion

This cross-sectional study characterizes levels of mental distress in Canada amid the COVID-19 pandemic, while examining the extent of virtual mental health resource use by level of mental distress and reasons for non-use. Findings identify a high burden of mental distress among adults living in Canada in the pandemic context (November to December 2021), with over half (51.2%) of survey respondents classified as experiencing moderate (36.6%) or severe (14.6%) levels of mental distress. These findings are consistent with a 2020–2021 Statistics Canada survey of 22,721 adults, in which 62.7% of the sample was found to have high levels of psychological distress, assessed using a related measure, the K10 Psychological Distress Scale.^34^ Moreover, these levels of mental distress are greater than what were seen in pre-pandemic assessments using the K6 scale, including a 2013 Canadian report that identified a 42.3% prevalence of moderate-severe mental distress among survey participants^35^ and another pre-pandemic report indicating that 6.5% of the general population in Canada had severe levels of distress.^36^ These data, together with our findings on virtual mental health resource use and non-use, offer critical direction for informing health communication strategies and evidence-informed responses to address population mental health during the pandemic and beyond.

Findings from this study underscore the ways in which perceptions about virtual mental health resource availability and appropriateness can influence their (non-)utilization. Indeed, this study identified a mismatch between survey respondents’ presumable need for mental health support (i.e. having moderate to severe levels of mental distress) and their use of virtual (and other) resources, including due to perceptions of this need (or the lack thereof) for help. Only one in five (19.3%) participants with moderate to severe mental distress indicated that they had used virtual mental health resources, with the most commonly reported reasons for not using these resources being that participants felt they did not need help (37.4%) and/or that they did not think the supports would be helpful (26.2%). These self-reported deterrents to service access are perhaps unsurprising, given that pre-existing beliefs about needing help are known to strongly influence mental health intervention engagement.^37^ Further, even if/when a person acknowledges a need for help and is willing to seek it, their decision about whether or not to do so can also be mediated by beliefs about intervention (in)effectiveness, particularly in the case of virtual mental health interventions.^37–39^ Still, it is noteworthy that uptake of virtual mental health resources was relatively low in this sample, as is consistent with our prior work on asynchronous virtual mental health resource uptake.^11^ This low uptake is intriguing when considering that the majority (82.2%) of survey respondents with moderate to severe distress knew that such services were available, derived from our finding that 17.8% of participants selected not knowing about the availability of these resources as a reason for not having used them. Moreover, this low uptake is salient given the escalating public health investment in virtual mental health^16,25^ and that other available supports (e.g. in-person services, peer networks) have been disrupted due to COVID-19 restrictions.^11,16,18,19^ Notwithstanding findings from our study with a nationally representative sample in Canada, work from other regions such as the United States has identified an expansion in both telehealth availability and adoption, including for mental health supports.^40,41^ In this context, there is a need for continued research that monitors trends in mental health resource use—inclusive of virtual but also in-person and hybrid supports—across time, regions, and populations, with explicit attention to tracking, and in turn, bridging gaps in resource availability and uptake, while also investigating who best responds to what types of virtual care.^25^

Findings from this study also offer directions for health communication related to the awareness and appropriateness of virtual mental health resources. The low prevalence of virtual mental health resource utilization use among survey respondents with moderate or severe mental distress in this study can again be partially explained by participants not perceiving a need for help (reported by 20.6% of participants with moderate distress and 44.6% of those with severe distress) and/or not thinking that virtual supports would be helpful (reported by 24.2% of participants with moderate distress and 30.0% of those with severe distress). This finding can be considered in connection to mental health literacy, a concept that is relevant to people across the full continua of mental health and illness and that refers to individuals’ “knowledge and beliefs about mental disorders which aid their recognition, management or prevention.”^42^ The population-level scope and relevance of mental health literacy is especially important to consider in the context of this study because, although virtual mental health resources are frequently recommended for people experiencing mild to moderate mental health challenges,^25^ this group was not using these resources to the extent that people with more severe mental distress were. There are many potential reasons for this, including for example that people with more severe mental distress may have a healthcare provider who had recommended the resource(s). Nonetheless, findings of this nature have valuable implications for focusing and refining health communications related to mental health literacy and virtual resource use. For instance, this study suggests a need for public health messaging that focuses on normalizing, validating, and de-stigmatizing experiences of mental distress, as well as messaging about the effectiveness and relevance of virtual resources for people with varying degrees of mental distress, including low-moderate distress. There may also be opportunities to tailor public health messaging to distinct population sub-groups, such as those most likely and/or unlikely to use virtual supports, about which our earlier study examined sociodemographic (e.g. age, geography) and health-related characteristics (e.g. receiving in-person mental health supports) associated with virtual mental health resource use amid COVID-19.^11^ Although health communication was not our study focus, there is an established literature on interventions to facilitate acceptance of health behaviors and services (including in the context of mental health), with these interventions often involving multi-pronged information strategies.^43,44^ Promising strategies for promoting virtual mental health resource uptake could include, for example, the provision of simple, context-sensitive information about intervention applicability and credibility, such as through offering testimonials from previous service users and experts, as was shown to be effective in a recent study on digital mental health services with college students.^45^ More generally, our study findings underscore the role of mental health literacy in shaping virtual resource access and utilization, and, in doing so, emphasize that fostering mental health literacy should be a key component of approaches to promote and protect population mental health.^42,46^

This study substantiates the literature on how service-user preferences for care delivery modalities (e.g. in-person versus virtual) and time-, privacy-, and stigma-related concerns can influence virtual mental health resource access and uptake.^24,37,47^ A considerable portion (59.9%) of surveyed participants with moderate and severe mental distress indicated they had not accessed virtual mental health resources. Reasons for not doing so included preferring in-person supports (23.4%), having competing time demands (14.0%), privacy concerns (10.9%), and stigma (8.0%). These reasons align with those documented in a recent systematic review,^37^ which identified a service-user preference for virtual mental health resources to be used as a complement, not a replacement, to existing in-person supports.^25,39,48–53^ This view is largely shared in the (digital) mental health field; from our perspective, virtual supports should be one component of a multi-pronged approach to mental health promotion and care, inclusive of interventions across socio-ecological domains, as we detail elsewhere.^46,54,55^ Still, targeted public health messaging could help to alleviate concerns individuals may have about the utility of virtual mental health resources whilst promoting their unique benefits, such as the potential for anonymity, low (or no) cost, and an enhanced sense of self-directedness and control in service uptake.^11,37,47^ Despite these benefits, our study and several of those in Borghouts and colleagues’ review^37^ found that engagement with digital mental health interventions is negatively affected by service users lacking time,^10,56–58^ interventions taking up too much time,^48,59,60^ and lack of a private space to use the interventions.^61–63^ Many of these issues have been exacerbated by the COVID-19 pandemic, which brought additional stressors and time and space constraints, with many people now working/studying from home (often in busier households) with potentially limited access to regular supports, including family members and childcare.^47^ This contextual issue relates to the privacy and stigma concerns surfaced in our study, concerns that were especially prevalent in participants with severe mental distress. Importantly then, although virtual service delivery may offer a boon toward mitigating some stigma and privacy concerns,^24^ our study identified that these issues persist and continue to shape intervention utilization. Factors such as these must be closely attended to when developing and evaluating service delivery in this sphere,^37,47^ whilst ensuring that virtual interventions are implemented in ways that are successful, impactful, and equitable.

### Limitations

There are strengths and limitations to this study. The sampling approach resulted in a large sample that is nationally representative according to age, gender, annual household income, and province/territory. Yet, the sample may be less representative according to other demographic characteristics, such as ethnocultural identity and dis/ability. Despite high levels of internet usage in Canada,^26^ there is also the potential for sampling bias given this is an online survey and only those individuals with internet access were able to participate. Indeed, individuals who were unable to access internet or other necessary technology for virtual mental health resource engagement, as well as those living in rural “blackout zones,” are likely under-represented in the sample. This may have influenced our study findings about internet and technology barriers to virtual mental health resource utilization, including that few participants with moderate to severe mental distress reported internet connectivity issues (3.9% of participants) and poor access to audiovisual equipment (2.8%) as reasons for not using virtual mental health resources. These are important equity issues to account for because limited access to technology/internet and related equipment is known to hamper virtual mental health resource delivery and uptake, especially in lower-resource and rural settings, and with people of lower socio-economic status.^37,64^ Related to this, the determinants or “reasons” for not using virtual mental health resources identified in this study are limited to those represented in the survey items; future work should seek to explore this issue more comprehensively, including by adding measures to capture other considerations, such as gaps in technological knowledge and competencies. Here, there are also opportunities for more targeted research on mental health resource use among people who are experiencing distress yet not accessing pertinent services (whether virtual, in-person, or neither).

This study directed limited attention toward potential inequities in experiences of mental distress and virtual mental health resource use. This is a key study limitation and an area for more targeted, equity-focused research. To this point, our team has elsewhere investigated the differential impacts of the COVID-19 pandemic on populations who experience intersecting structural vulnerabilities,^46,54,55^ including in the context of virtual mental health resource uptake.^11^ In this survey, we also note that a considerable subset (*n*  =  315) of respondents experiencing moderate to severe mental distress indicated, “Not applicable. I haven’t experienced a mental health challenge during the pandemic” when asked about their use of virtual mental health resources. However, the vast majority of these respondents were people experiencing moderate distress, among whom many bordered on low distress, as is reflected in Figure 1. Indeed, respondents with higher levels of moderate distress tended to endorse having a need for mental health support. As with any survey about sensitive, stigmatized phenomena, there is also potential for self-reporting and social desirability bias in this study. Finally, the cross-sectional study design prevents us from drawing causal conclusions and also limits our ability to examine participants’ virtual mental health resource utilization over time. Future research using longitudinal methods and qualitative or mixed-methods designs may generate additional insights into population mental health and virtual mental health resource use (and non-use) now and throughout future phases of the pandemic.

## Conclusion

Virtual mental health resources offer a valuable means of promoting and protecting mental health among people experiencing or “at-risk” of mental health challenges, including in the context of the COVID-19 pandemic and associated stressors. These resources may be especially applicable to people experiencing high, or heightened, levels of mental distress during the pandemic, as well as those facing disrupted access to traditional supports, such as in-person services. Unfortunately, this study identified that despite there being high levels of mental distress among adults living in Canada during the pandemic, uptake of virtual mental health resources remained relatively low. This was due to a range of reasons, with study participants experiencing mental distress most commonly reporting not feeling as though they need help, not thinking the supports would be helpful, preferring in-person supports, and not knowing the supports were available. Non-access to supports was also due to competing time demands and stigma and privacy concerns. Taken together, this study's findings on the extent of virtual mental health resource use (and non-use) offer important directions for focusing and refining health communication in relation to mental health literacy and the awareness and appropriateness of virtual mental health resources. Specifically, there is a need for public health messaging that focuses on normalizing and validating experiences of mental distress, as well as messaging about the effectiveness and relevance of virtual mental health resources for people across the full continua of mental health and illness, above and beyond messaging that merely informs people of the existence of these services. More generally, this study underscores the potential for multi-pronged approaches to mental health promotion during the COVID-19 pandemic and beyond, inclusive of virtual, in-person, and upstream policy interventions and supports.
